# Assessing Learning Needs and Career Attitudes of Italian Psychiatry Residents: Results from a National Survey Conducted by the Italian Society of Psychopathology Young Psychiatrists Section (SOPSI-GG)

**DOI:** 10.2174/1745017901915010021

**Published:** 2019-02-20

**Authors:** Stefano Barlati, Massimiliano Buoli, Annabella Di Giorgio, Giorgio Di Lorenzo, Carla Gramaglia, Eleonora Gattoni, Andrea Aguglia, Alessio Maria Monteleone, Bernardo Dell’Osso

**Affiliations:** 1 Department of Clinical and Experimental Sciences, University of Brescia, Brescia, Italy; 2 Department of Psychiatry, University of Milan, Fondazione IRCCS Ca'Granda Ospedale Maggiore Policlinico Milan, Italy; 3 IRCCS "Casa Sollievo della Sofferenza", San Giovanni Rotondo, Foggia, Italy; 4 Department of Systems Medicine, University of Rome "Tor Vergata", Rome, Italy; Psychiatry and Clinical Psychology Unit, Department of Neurosciences, Fondazione Policlinico "Tor Vergata", Rome, Italy; 5 Department of Translational Medicine, Institute of Psychiatry, Università del Piemonte Orientale, Novara, Italy; 6 Department of Neuroscience, Rehabilitation, Ophthalmology, Genetics Maternal and Child Health, University of Genoa, Psychiatry Unit, “IRCCS Policlinico San Martino” Hospital, Genoa, Italy; 7 “Rita Levi Montalcini” Department of Neuroscience, Turin, Italy; 8 Department of Psychiatry, University of Campania "Luigi Vanvitelli", Naples, Italy; 9 Department of Biomedical and Clinical Sciences “Luigi Sacco”, University of Milan, ASST Fatebenefratelli-Sacco, Milan, Italy; 10 Department of Psychiatry and Behavioral Sciences, Stanford University, Stanford, CA, USA; 11 Institute of Psychiatry, Maggiore della Carità Hospital of Novara, Novara, Italy

**Keywords:** Psychiatry, Residency, Education, Training, Learning needs, SOPSI-GG

## Abstract

**Objective:**

No data are available about learning needs and career attitudes of Italian Psychiatry Residents (IPRs). Authors aimed to assess such needs through a survey to generate insight for implementing educational programs close to IPRs’ perceived learning needs.

**Methods:**

A 54-item questionnaire was developed in order to investigate career information, educational preference and learning needs of IPRs. A sample of 298 IPRs participated to the survey and was divided into four subgroups according to their location (North, Centre, South and Islands). The subgroups were compared through ANOVA for age and chi-square tests for qualitative variables (including gender and all sub-items of the survey), with Bonferroni post-hoc analysis.

**Results:**

IPRs were found to pursue, along with traditional and theoretical training, a quite practical approach, characterized by working groups, discussions on clinical cases and practical interactive sessions. The topics of major interest included: clinical psychiatry, psychopharmacology, psychiatric emergencies, communication and relationship skills (97%, 98.0%, 98.3% and 95.7% of the total sample, respectively). Indeed, a strong need for interaction with healthcare professionals emerged (97% of the total sample). North and Centre IPRs were more involved in Day Hospital activities than residents from South Italy and Islands (p<.001). South IPRs appeared to be more prone to invest for their education than residents from other areas (*p*<.01).

**Conclusion:**

Reported findings should be taken into account as a starting point for planning and developing future targeted packages of educational proposals for IPRs and they should stand as a useful pilot study for further investigation in the field.

## 1 INTRODUCTION

Continuous education is warranted in every medical field (including psychiatry), at any time, over clinicians’ career. In particular, training, education and learning represent the nuclear components of residents’ professional life. Residents’ training and education should encompass all the following three areas: theoretical knowledge (know that and know what), practical knowledge (knowhow and technical skills), and attitude (one's relational skills). Actually, teaching methods across residency schools may be extremely different. Besides traditional methods (*e.g.*, lectures), more “informal” approaches include journal clubs, problem-based learning, formalized patient-centered training, and games [1]. Teaching methods may be boosted by the use of modern technology, with the development of new training possibilities, such as web-based approaches, simulation, and movies [2-10].

In Italy, the Ministerial Decree (Gazzetta Ufficiale 258, November 5^th^, 2005) “Reorganization of Medical Residency Training Program” defined common planning for medical training in every medical residency school and provided a standard approach for psychiatry as well. This plan provides a core curriculum and elective characterizing activities chosen by the resident. The core curriculum includes basic training objectives, such as anatomophysiology of the nervous structures, biochemical, physiological, endocrinological and neuroradiological correlates of psychiatric syndromes; principles of genetic research in psychiatry; skills training in the field of general psychopathology; use of diagnostic and evaluation tools; adequate interpretation of differential diagnostic profiles and rational use of therapies and drugs; skills training of clinical psychiatry with knowledge of the diagnostic, clinical and prognostic characteristics of psychiatric diseases of the young, adult and advanced age, personality disorders, drug-addictions and psychosomatic illnesses; technical and methodological skills necessary to deal with different clinical situations, including crisis and liason psychiatry. The elective characterizing activities are mainly in-depth studies in the areas of forensic psychiatry, addiction, geriatric psychiatry, adolescent psychiatry, liason psychiatry, eating disorders and psychiatric rehabilitation. Nonetheless, an evidence-based approach for psychiatry residents training, including the assessment of learning needs and the evaluation of educational curricula, highlights that course contents and outcome measures widely vary across studies, making literature reports inconsistent and difficult to compare. For instance, some studies have been focused on a specific field of psychiatric education (*e.g.*, community psychiatry, suicide, ethics, tele-psychiatry, eating disorders, substance use disorders, psychotherapy), often with locally developed surveys for the assessment of residents’ learning needs [11-17].

Every year, about 218 new Italian psychiatry residents (IPRs) are recruited, and psychiatry residents are currently almost 900 professionals attending a 4 years post-graduate training (for residents who took up their career after 2014, 5 years before 2014). The first year usually includes 3 months of Internal Medicine and the following years, neurology and child psychiatry training. In addition, the trainees have to participate in educational (*e.g.* class) and research activities. Trainees are usually solicited to follow some cases in psychotherapy and some cases of patients suffering from substance use disorder. The quality of psychiatry residency program is guaranteed by the Ministry of Education and the different Universities. However, according to the autonomy of the single Universities and within a framework of minimally guaranteed activities, residents from different parts of Italy may follow a slightly different training path depending on the place. The training network, therefore, depends on each local possibility and this contributes to increase the heterogeneity in this field. A similar organization of the psychiatry residency program is found in other countries, such as Canada, while training programs tend to be more homogeneous in the United States or in South Korea [18]. In countries such as Chile, critical issues for residency schools include that psychiatry residents are not paid for their clinical work and the limited access to electronic journal for most universities. In Sweden, residents are addressed prematurely to full clinical work for lack of specialists [18]. Of note, in Italy, IPR’ salary is paid by the University, differently from other European countries, where they already work as employees of the National Health Service.

The existing literature about residents’ learning needs is still scant worldwide and, to date and to our knowledge, no assessment of IPRs’ learning needs and career attitudes has been performed. Current training for psychiatry residents is highly variable in terms of methodology and contents, with relevant differences from one country to another, and within the same country, according to the specific orientation and tradition, characterizing each residency school [19]. More specifically, the different approaches towards psychiatry teaching in Italian residency schools often tend to be influenced by local priorities of research and scientific interests, with some schools following a more biologically oriented approach, and others a more psychosocial one, using different teaching and training methods. As a consequence, residents may not obtain a standard set of knowledge and professional skills at the end of their residency, with some of them following parallel integrative private pathways of learning /training (*e.g.*, in relation to psychotherapy).

Even though all the above can produce some gap in residents’ education, it is difficult to draw clear conclusion in the field, as specific reports are lacking. In addition, it needs to be taken into account that differences in training methods across Italian psychiatry residency schools may also be related to geographic factors, as the country may be divided in 4 macro-areas (Northern Italy, Centre Italy, Southern Italy, Islands). Therefore, the aim of the present study was to assess and compare learning needs and career attitudes of IPRs (under the age of 40 years) across the aforementioned macro-areas, using a specifically developed questionnaire in order to highlight potential differences and possible targets needing further enhancement in terms of training and education.

## 2 METHODS

The study proposal emerged in the context of a SOPSI-GG initiative (SOcietà Italiana di PSIcopatologia-Gruppo Giovani, Italian Society of Psychopathology - young psychiatrists section). The group was born in 2015 during the annual SOPSI Meeting (Milan, February 2015) with the aim to enhance scientific collaboration and education and promote scientific and research activities of young Italian psychiatrists and residents in Psychiatry.

### 2.1 Assessment: Residents’ Learning Needs Questionnaire

Authors developed a questionnaire for the assessment of young Italian psychiatrists’ learning, education and training needs. Focusing on the present study, it was administered only to residents in training, as homogeneous population and principal target of this investigation. It was designed as a 54-item, structured, anonymous, survey questionnaire (see Appendix 1). The questionnaire consisted of three sections. The first section included 6 items and was aimed to collect residents’ features, as they represent the target of training and education activities. The second section consisted of 3 items, aimed to investigate the preferred methods for learning and training delivery. Finally, the third section included 43 items, aimed to identify the main areas of interest and the factors that mostly influence the participation to formative events. The last questions assessed the needs of young residents to deal with other psychiatrists and colleagues (for instance, operators of Mental Health Services and/or clinicians of other medical fields), leaving room for suggestions and comments. Furthermore, two additional questions were included to obtain demographic data (age and gender) of participants.

Each of the three main sections included closed questions, some encoded according to a Likert 5-point scale (1 = not at all important, 5 = extremely important) and others in the format of multiple choice.

The theoretical basis underlying the questionnaire was an integrated approach to psychiatric care, spanning from clinical and psychopharmacological issues to boundary areas, forensic and organizational aspects, research and rehabilitation programs.

The questionnaire was developed following some available models in the scientific literature, related to personal satisfaction [20], and adapted to the current educational plans of the Italian residency schools in Psychiatry. Particular attention was also paid to the recent literature about the most innovative specialized care approaches [21].

The questionnaire was then submitted for review to a team (n=26) of psychiatrists and residents in Psychiatry (from the directory boards of SOPSI-GG and SOPSI) and then it was circulated by mail to the 36 secretariats of the Italian residency schools in Psychiatry in order to be distributed among residents.

### 2.2 Statistical Analyses

With respect to the calculation of the sample size, the total number of IPRs corresponded to approximately 872 subjects (218 positions each year) and a confidence interval of 5 was expected with regard to age (corresponding to the years of psychiatry residency). In this case, for a statistical significance < .05 or, alternatively, a Cohen’s d effect size of at least 0.4 for the main included variables (*e.g.* current workplace), a sample of 267 subjects was considered of adequate power. Descriptive analyses of the total sample were performed. The subgroups (divided according to the following geographical areas: North, Centre, South and Islands) were compared through univariate Analysis Of Variance (ANOVA) for age and chi-square tests for qualitative variables, with Bonferroni as post-hoc analysis (significance of post-hoc analyses were adjusted for multiple comparisons according to Bonferroni’s correction). Qualitative variables included gender and all sub-items of the survey. Statistical Package for Social Sciences (SPSS) for Windows (version 22.0) was used as a statistical analysis program [22].

## 3 RESULTS

The sample included 298 participants: 107 males (35.9%) and 191 females (64.1%), with a mean age + SD (standard deviation) of 30.58 + 3.83 years (Northern Italy: 30.37 + 3.49, Centre Italy: 30.66 + 3.67, Southern Italy: 30.60 + 4.15, Islands (30.82 + 3.68). The response rate was 34% and no questionnaire was returned incomplete. Northern Italy (N=72) included the Universities of the following cities: Milan, Brescia, Novara, Padua, Turin; Centre Italy (N=48): Chieti, Rome, Siena; Southern Italy (N=134): Bari, Foggia, Naples; Islands (N=44): Cagliari (Sardinia), Catania, Messina and Palermo (Sicily).

### 3.1 Current Occupation

Descriptive statistics showed that most of the IPRs work in University-affiliated Clinics/services (82.2%). When the type of service was considered, inpatient clinics were found to be the most common workplace for IPRs (56.6%). Of note, 167 IPRs were currently working in inpatient clinics, 32 in outpatient clinics, 2 in a psychiatric residential facility, 25 in a psychiatric semi-residential facility, 13 in an Eating Disorder Centre, 59 in a Day Hospital service.

With the exception of adult psychiatry, the most clinically relevant area of work for residents covered patients suffering from substance abuse (15.8% of total sample) followed by patients with social problems (*e.g.*, homeless or immigrants) (3.4%) and with neurodegenerative disorders (3.0%).

### 3.2 Education

In terms of educational opportunities, psychiatry residents were found to prefer to attend congresses (61.4%) and discuss practical clinical cases (68.8%), particularly when these activities are organized during working time (72.1%). The most important factors in selecting educational opportunities were represented by: qualification of trainers (97.6%), educational methods (90.6%), available lectures (87.2%) or tutorials in small groups (77.9%), and economic costs (70.8%). The most interesting topics for training were represented by: psychiatric emergencies (98.3%), Psychopharmacology (98.0%), clinical psychiatry (97%), management of special populations (96.6%) (*e.g.*, pregnant patients or elderly subjects with psychiatric disorders), suicidal behaviours (96.0%) and psychiatric-patient relationship (95.7%). On the other hand, topics considered to be less important for training by psychiatry residents included: the organization of psychiatric services (69.1%), child psychiatry (75.2%) and community psychiatry (78.5%) (percentages are referred to subjects who marked the topic as of interest). Finally, almost all residents showed a specific interest toward interactions with other psychiatrists (98.3%) and healthcare professionals (97%).

Figs. (**1** and **2**) report satisfaction rates of psychiatry residents about different teaching methods and rates of interest of trainees in relation to the different topics, respectively.

### 3.3 Group Comparisons (Macro-Areas)

Subgroups divided according to geographical areas were not significantly different in terms of age (F=0.12, *p*=.95), gender distribution (χ^2^=4.45, df=3, *p*=.21) and years of training (χ2=7.99, df=6, p=.23).

IPRs showed significant differences in terms of the workplace (χ^2^=24.23, df=15, *p*=.048): trainees from Islands were found to work less frequently in community health services than those from other areas (*p*<.05). Similarly, they reported to carry out their clinical activities in different settings (χ^2^=181.19, df=18, *p*<.001). For instance, being involved in Day-Hospital activities was more frequent in Centre than in Northern Italy (*p*<.05), but less common in Southern Italy and Islands than in Northern Italy (*p*<.05). In addition, trainees from Northern Italy reported to spend their work time in multiple psychiatric structures (*e.g.*, Clinics with outpatient and inpatient units) more frequently than residents from other geographical areas (χ^2^=11.64, df=3, *p*=.01), and to be more involved in child psychiatry services than trainees from other areas (*p*<.05). Geriatric patients (χ^2^=11.35, df=3, *p*=.01) and those with substance abuse (χ^2^=16.33, df=8, *p*=.03) were found to be more frequently managed by residents from Islands than trainees from other areas, while adolescents were found to be less frequently managed by residents from Islands than trainees from other regions (χ^2^=18.06, df=9, *p*=.03).

With regard to the type of training, workshops were found to be more appreciated by residents from Islands than those from other districts (χ^2^=14.86, df=3, *p*=.002). With regard to educational methods, round tables were less appreciated by trainees from Centre Italy than those from other areas (χ^2^=8.25, df=3, *p*=.04). Clinical case discussion was more valued by residents from Centre Italy than those from other regions (χ^2^=10.44, df=3, *p*=.01), while small group workshops were less appreciated by trainees from Centre Italy than those from other areas (χ^2^=7.58, df=3, *p*=.05). In addition, video conferences were more despised by residents from Northern Italy than those from other areas (χ^2^=9.12, df=3, *p*=.03). Trainees from Southern Italy were more prone to invest money for their education than residents from other areas (χ^2^=31.89, df=15, *p*=.004), while trainees from Islands considered the University sponsorship (χ^2^=29.42, df=15, *p*=.01), but not the qualification of tutors (χ^2^=25.15, df=15, *p*=0.03), as a basic requirement for the selection of educational opportunities. The type of institution (*e.g.* a scientific society or university) responsible for the training organization (χ^2^=34.77, df=15, *p*=.002) and methods (χ^2^=45.80, df=15, *p*<.001) was, moreover, considered less important in the choice of educational events by residents from Centre Italy than those from other areas, while the need for overnight (χ^2^=26.01, df=15, *p*=.04) and the lack of public transport (χ^2^=32.57, df=15, *p*=.003) were seen as obstacles to the participation to educational events more by trainees from Southern Italy than those from other areas. Finally, trainees from Northern and Centre Italy were found to prefer educational events taking place during working time (χ^2^=29.59, df=15, *p*=.01). With regard to the topics covered during psychiatry residency program, the psychiatrist-patient relationship seemed to attract more interest from residents from Northern Italy than those from other areas (χ^2^=28.43, df=15, *p*=.02), while trainees from Islands reported less interest in local (χ^2^=27.45, df=15, *p*=.02) and community psychiatry (χ^2^=31.58, df=15, *p*=.01) than residents from other areas. In addition, special populations (*e.g.*, pregnant women with psychiatric disorders) were found to be more studied by trainees from Centre Italy than those from other areas (χ^2^=26.02, df=15, *p*=.03), while child psychiatry (χ^2^=31.76, df=15, *p*=.01) and psychiatric aspects of neurodegenerative disorders (χ^2^=25.94, df=15, *p*=.03) attracted less frequently the interest of residents from Northern Italy than those from other areas.

Fig. (**3**) shows factors involved in selecting educational opportunities by IPRs for the four selected Italian geographical areas.

## 4 DISCUSSION

To our knowledge, this is the first study exploring and comparing learning needs, preferences and career attitudes of IPRs grouped according to different geographic areas. In this survey, the response rate for residents was 34%, slightly lower compared to what previous international studies in the field reported, ranging from 40.5% to 86% [14, 18, 20, 23, 24]. This response rate might be due to several factors, such as the (only) recent launch of the SOPSI-GG section, with a limited power to reach residents and poor attitude to research on this topic in Italy.

The overall results showed that residents are highly interested in being trained and improving their knowledge and professional skills, as underlined by the high response rate to the likert item “very important” and “extremely important” on the questionnaire section: *“How much do the following topics meet your training needs?”*.

With respect to factors influencing the choice for their training within the educational programs, residents considered of primary importance the qualification of trainers. This finding represents a crucial point for the implementation of training courses, suggesting to take into account trainers’ *curricula* and related skills and expertise.

In addition, the training method was found of crucial importance by residents. Along with traditional education methods, such as congress sessions and lectures, residents showed to prefer a more practical approach, characterized by working groups, discussions of clinical cases and interactive sessions (*e.g.*, workshop, role play, *etc*.), especially when these events are organized during working time. This is in line with a study performed by Sockalingam and colleagues (2008) in which more than 50% of residents in psychiatry reported to prefer interactive pedagogical methods, including workshops, small groups, mentoring, and didactic learning methods in order to improve their knowledge and skills [25]. As evidenced by the international literature on this topic, these preferences refer to the concepts of theoretical and practical knowledge (know that and knowhow), as essential parts of the training process [26, 27]. Furthermore, the knowhow and attitude, defined as the way of behaving, thinking and feeling about something, seemed to be necessary for the development of the career, even in light of their related emerging issues of major interest. Indeed, psychopathological and clinical issues, psychopharmacological knowledge and capacity to manage psychiatric emergencies - all topics that refer to the concepts of know that and knowhow - emerged as the most important in relation to learning for psychiatry residents.

Besides these topics, residents would also like to be trained and updated on the most effective strategies for managing the relationship with the patient, with particular emphasis on clinical interviews and therapeutic alliance. Therefore, it comes into play the third essential component of the learning process: attitude [26, 28]. Based on these considerations, it seems crucial to plan future learning events integrating both traditional and theoretical issues with more practical elements closer to every day clinical practice.

Less remarked than previous issues, but nevertheless underlined among the factors influencing residents’ choice within different training paths, was the economical aspect. In this regard, overall costs should be taken into account in planning educational events, providing a budget and/or specific facilities for residents’ training, encouraging them to join scientific societies and related benefits. This could lead not only to obtain discounted congress registrations but also to be connected with other professionals, building an effective network collaboration. This approach could also stimulate the development of a cultural context, which strongly values residents’ training and personal responsibility in terms of extra-curricular education.

Of note, residents reported a marked need of interaction and comparison with other residents in Psychiatry and psychiatrists as well as with other professionals working in the field of mental health and/or clinicians of other disciplines. For these reasons, in planning vocational training, it should be taken into account to provide multidisciplinary, theoretical and practical updates, addressed to different professional categories. This would allow a continuous exchange and interaction between different operators, following the fundamental principles of the integrated care in psychiatry: working in a multidisciplinary team and integrating different expertise when planning and providing therapeutic interventions [29, 30].

Despite the current technological time, in which Internet appears to be the main form of communication and transmission of knowledge, especially among young people, online courses and e-learning were not found to be the preferred methods of training delivery. This is of particular interest and may mirror the importance attributed to personal relationships in terms of education and training [26].

The sub-analysis of the four different Italian geographical areas (North, Centre, South and Islands) reported mixed data, with specific trends and local differences. In particular, with regard to the type of training, the discussion of clinical cases was more valued by residents from Centre Italy, while workshops were found to be more appreciated by residents from Islands. Furthermore, in relation to factors influencing the educational choices, trainees from Northern and Centre Italy were found to prefer educational events during working time, trainees from Southern Italy were more prone to spend money for their education, while trainees from Islands preferred educational events, organized and promoted by their University. With regard to the topics covered during residency program, the psychiatrist-patient relationship was the most interesting issue for residents from Northern Italy, while special populations (*e.g.*, pregnant women with psychiatric disorders) were found to be the preferred topic by trainees from Centre Italy.

The observed variability of learning needs across specific geographical areas clearly indicates the lack of a standardized training approach for psychiatry residency schools in Italy and likely confirms the existence of different training cultures and methods. Therefore, the interest expressed toward a specific topic may be linked either to perceived flaws in education (*i.e.*, to fill a perceived gap) or, on the other hand, to specific fields of interest of their residency school (*i.e.*, residents may try to further enhance their knowledge in that specific area). In any case, it will be useful and necessary to take into account these results to plan future training courses, in a more personalized perspective, corresponding to the real residents’ needs.

Taken as a whole, reported findings highlight not only the presence of several shared preferences in terms of learning needs but also many differences across Italian regions. In this regard, the programs of the Italian residency schools in Psychiatry should be more standardized and integrated to the real residents’ needs, promoting training courses and educational programs with standardized aims, contents and assumptions, including periods of exchange with other residency schools.

Despite the original findings that we discussed, this study has some limitations. In particular, although the sample was representative of the population under study, the questionnaire was not returned by all the IPRs. Therefore, one of the most important future goals, in order to enlarge the sample, will be to involve more participants into initiatives of this kind. Furthermore, the questionnaire represents a new tool, not previously validated, and developed to be applied to the contest of the Italian mental health care system. Nonetheless, its validation is currently in progress. Due to the profound differences in terms of organization, education system, and funding, the questionnaire has not been translated for use in countries other than Italy yet. Another limit is the difference in sample size between the geographical areas, but this is at least in part representative of the Italian population distribution: Southern Italy has more inhabitants than Centre Italy and one region of Southern Italy (Campania) has the highest population density in Italy. Other confounding factors are more difficult to evaluate in light of the different clinical needs of the territory where residents make their clinical practice. It is more difficult to believe - but it cannot be excluded - that residents’ interest is driven by the lack of specific facilities. It is more difficult to believe - but it cannot be excluded - that residents’ interest is driven by the lack of specific facilities. In particular, it could be hypothesized that residents might manage specific types of patients reflecting specific contexts like local diffusion of illicit drugs or heterogeneity in family support in rural versus urban areas.

## CONCLUSION

In conclusion, this study underlined that IPRs are particularly motivated to be trained and to update their knowledge, improving skills in their daily clinical practice. Main results highlight the preference towards specific topics and methods of training delivery, including more practical approach characterized by working groups, discussions of clinical cases and practical interactive sessions, particularly when they are organized during working time. The main identified training axes were: 1) psychopathological and clinical issues, 2) psychopharmacological knowledge, 3) psychiatric emergencies, and 4) communication and relationship skills, in order to improve daily clinical practice. Among residents, there was a marked need of interaction and confrontation with colleagues of the same discipline and with other professionals working in the field of mental health and other medical areas, with the aim to promote collaboration and interactive discussion. The results of this survey should be taken into account as a starting point for planning and developing future targeted packages of educational proposals for IPRs. We expect that these findings should stand as a useful pilot study for further investigation in the field.

## Figures and Tables

**Fig. (1):**
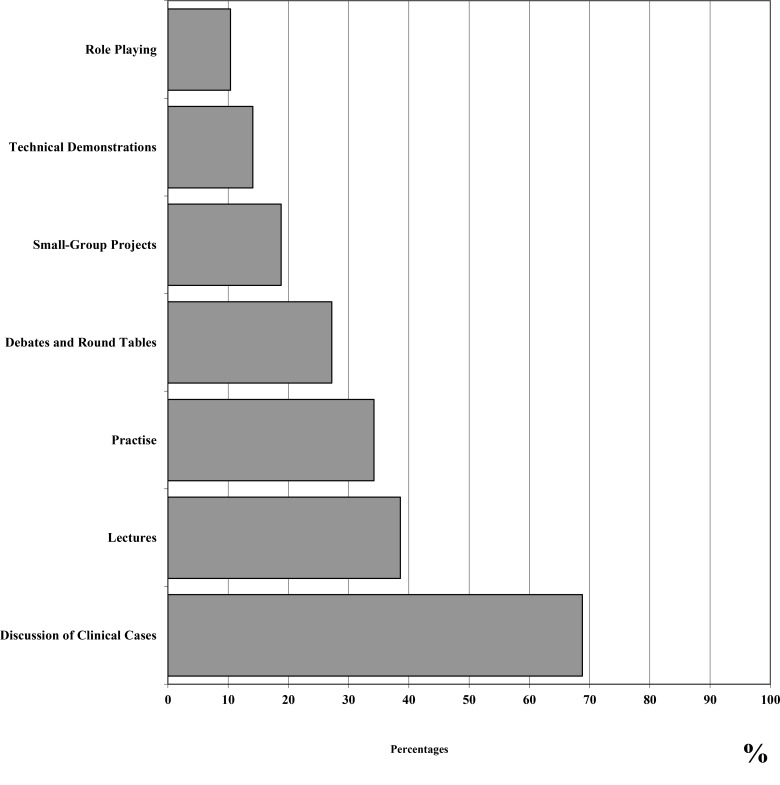
Percentage of satisfaction of psychiatry residents about the different teaching methods.

**Fig. (2):**
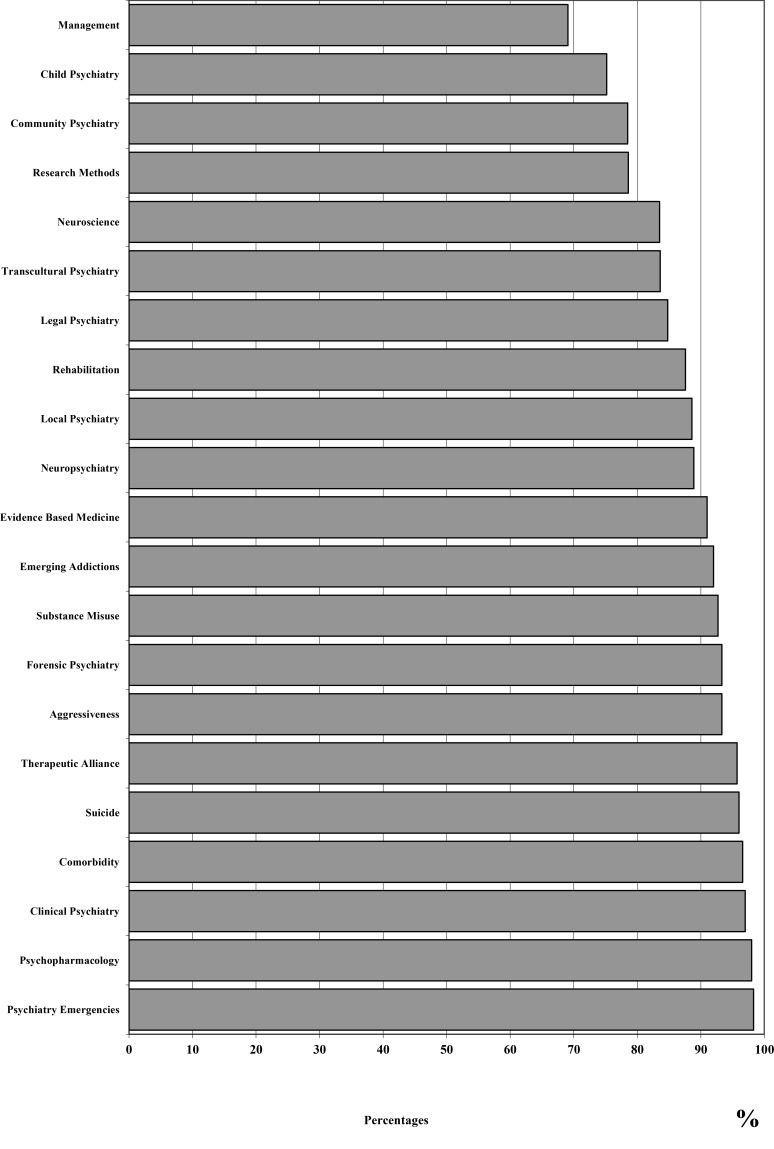
Percentages of interest of trainees with regard to the different psychiatry topics.

**Fig. (3):**
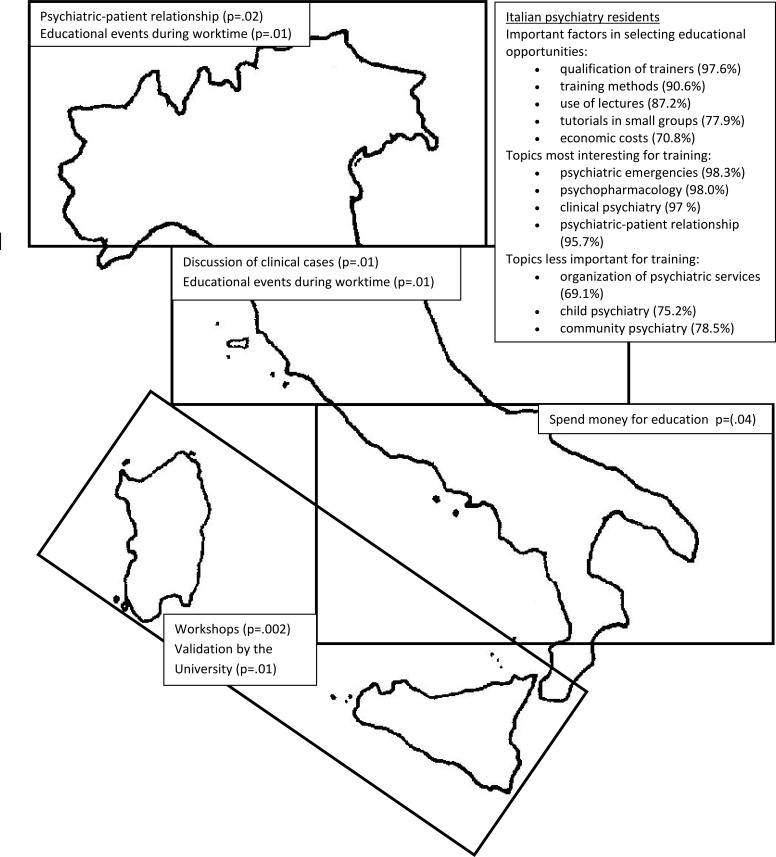
Factors involved in selecting educational opportunities for the four Italian geographical areas (North, Centre, South and Islands).

## References

[1] Zisook S., Benjamin S., Balon R., Glick I., Louie A., Moutier C., Moyer T., Santos C., Servis M. (2005). Alternate methods of teaching psychopharmacology.. Acad. Psychiatry.

[2] Gramaglia C., Jona A., Imperatori F., Torre E., Zeppegno P. (2013). Cinema in the training of psychiatry residents: focus on helping relationships.. BMC Med. Educ..

[3] Johnson J.M., Beresin E.V., Stern T.A. (2014). Using Breaking Bad to teach about defense mechanisms.. Acad. Psychiatry.

[4] Sierles F.S. (2005). Using film as the basis of an american culture course for first-year psychiatry residents.. Acad. Psychiatry.

[5] Torre E. (1999). La Psichiatria di Liaison: il modello e la relazione.. Psichiatria di Consultazione..

[6] Torre E., Usai C., Torre E.M.T., Ponzetti D., Gramaglia C., Marangon D., Zeppegno P. (2017). Educating to helping relationships: an innovative approach with the use of movies.. Film and Cinema: Past, Present and Future Perspectives..

[7] Vestal H.S., Sowden G., Nejad S., Stoklosa J., Valcourt S.C., Keary C., Caminis A., Huffman J. (2016). Simulation-Based Training for Residents in the Management of Acute Agitation: A Cluster Randomized Controlled Trial.. Acad. Psychiatry.

[8] Wilkening G.L., Gannon J.M., Ross C., Brennan J.L., Fabian T.J., Marcsisin M.J., Benedict N.J. (2016). Evaluation of Branched-Narrative Virtual Patients for Interprofessional Education of Psychiatry Residents.. Acad. Psychiatry.

[9] Zeppegno P., Gramaglia C., Feggi A., Lombardi A., Torre E. (2015). The effectiveness of a new approach using movies in the training of medical students.. Perspect. Med. Educ..

[10] Zeppegno P., Gramaglia C., Torre E.M.T., Feggi A., Marangon D., Usai C., Torre E., Copeland J. (2017). A cineforum project for medical students: learning from unexpected cues.. Film and Cinema: Past, Present and Future Perspectives..

[11] Avery J., Zerbo E. (2015). Improving Psychiatry Residents’ Attitudes Toward Individuals Diagnosed with Substance Use Disorders.. Harv. Rev. Psychiatry.

[12] Barekatain M., Aminoroaia M., Samimi S.M., Rajabi F., Attari A. (2013). Educational needs assessment for psychiatry residents to prevent suicide: a qualitative approach.. Int. J. Prev. Med..

[13] Goldman C.R., Brown D.B., Thompson K.S. (1993). Community psychiatry training for general psychiatry residents: results of a national survey.. Community Ment. Health J..

[14] Kovach J.G., Dubin W.R., Combs C.J. (2015). Psychotherapy Training: Residents’ Perceptions and Experiences.. Acad. Psychiatry.

[15] Roberts L.W., Warner T.D., Hammond K.A., Geppert C.M., Heinrich T. (2005). Becoming a good doctor: perceived need for ethics training focused on practical and professional development topics.. Acad. Psychiatry.

[16] Sunderji N., Crawford A., Jovanovic M. (2015). Telepsychiatry in graduate medical education: a narrative review.. Acad. Psychiatry.

[17] Williams M., Leichner P. (2006). More training needed in eating disorders: A time cohort comparison study of Canadian psychiatry residents.. Eat. Disord..

[18] Zisook S., Balon R., Björkstén K.S., Everall I., Dunn L., Ganadjian K., Jin H., Parikh S., Sciolla A., Sidhartha T., Yoo T. (2007). Psychiatry residency training around the world.. Acad. Psychiatry.

[19] de la Garza S., Phuoc V., Throneberry S., Blumenthal-Barby J., McCullough L., Coverdale J. (2017). Teaching Medical Ethics in Graduate and Undergraduate Medical Education: A Systematic Review of Effectiveness.. Acad. Psychiatry.

[20] Sockalingam S., Wiljer D., Yufe S., Knox M.K., Fefergrad M., Silver I., Harris I., Tekian A. (2016). The Relationship Between Academic Motivation and Lifelong Learning During Residency: A Study of Psychiatry Residents.. Acad. Med..

[21] Lobo A. (2015). de-la-Cámara, Campos R, Ventura T, Marco C, Campayo A, Dourdil F, Fé Barcones M, Saz P. Innovative methods in teaching psychiatry to medical students.. Eur. J. Psychiatry.

[22] IBM Corp IBM SPSS Statistics for Windows, Version 22.0.

[23] Grujich N.N., Razmy A., Zaretsky A., Styra R.G., Sockalingam S. (2012). Evaluation of professional role competency during psychiatry residency.. Acad. Psychiatry.

[24] Rao N.R., Kodali R., Mian A., Ramtekkar U., Kamarajan C., Jibson M.D. (2012). Psychiatric residents’ attitudes toward and experiences with the clinical-skills verification process: a pilot study on U.S. and international medical graduates.. Acad. Psychiatry.

[25] Sockalingam S., Stergiopoulos V., Maggi J. (2008). Residents’ perceived physician-manager educational needs: a national survey of psychiatry residents.. Can. J. Psychiatry.

[26] Hofmann M., Harendza S., Meyer J., Drabik A., Reimer J., Kuhnigk O. (2013). Effect of medical education on students’ attitudes toward psychiatry and individuals with mental disorders.. Acad. Psychiatry.

[27] Sternberg R.J., Grigorenko E.L. (2003). The psychology of abilities, competences and expertise..

[28] Tiwari A., Lai P., So M., Yuen K. (2006). A comparison of the effects of problem-based learning and lecturing on the development of students’ critical thinking.. Med. Educ..

[29] Kane J.M., Robinson D.G., Schooler N.R., Mueser K.T., Penn D.L., Rosenheck R.A., Addington J., Brunette M.F., Correll C.U., Estroff S.E., Marcy P., Robinson J., Meyer-Kalos P.S., Gottlieb J.D., Glynn S.M., Lynde D.W., Pipes R., Kurian B.T., Miller A.L., Azrin S.T., Goldstein A.B., Severe J.B., Lin H., Sint K.J., John M., Heinssen R.K. (2016). Comprehensive Versus Usual Community Care for First-Episode Psychosis: 2-Year Outcomes From the NIMH RAISE Early Treatment Program.. Am. J. Psychiatry.

[30] Raine R., a′ Bháird C.N., Xanthopoulou P., Wallace I., Ardron D., Harris M., Barber J., Prentice A., Gibbs S., King M., Blazeby J.M., Michie S., Lanceley A., Clarke A., Livingston G. (2015). Use of a formal consensus development technique to produce recommendations for improving the effectiveness of adult mental health multidisciplinary team meetings.. BMC Psychiatry.

